# Machine learning models predicting risk of revision or secondary knee injury after anterior cruciate ligament reconstruction demonstrate variable discriminatory and accuracy performance: a systematic review

**DOI:** 10.1186/s12891-024-08228-w

**Published:** 2025-01-04

**Authors:** Benjamin Blackman, Prushoth Vivekanantha, Rafay Mughal, Ayoosh Pareek, Anthony Bozzo, Kristian Samuelsson, Darren de SA

**Affiliations:** 1 School of Medicine, University of Limerick, Limerick, Ireland; 2 Division of Orthopaedic Surgery, Department of Surgery, McMaster University, Hamilton, ON Canada; 3 Michael DeGroote School of Medicine, McMaster University, Hamilton, ON Canada; 4 Hospital for Special Surgery, New York, NY USA; 5 McGill University Health Center, Montreal, QC Canada; 6 Department of Orthopaedics, Institute of Clinical Sciences, Sahlgrenska Academy, University of Gothenburg, Göteborg, Sweden; 7 Department of Orthopaedics, Sahlgrenska University Hospital, Mölndal, 431 80 Sweden

**Keywords:** Machine learning, AI, Anterior cruciate ligament, Revision, Reoperation, Modeling, Arthroscopy

## Abstract

**Background:**

To summarize the statistical performance of machine learning in predicting revision, secondary knee injury, or reoperations following anterior cruciate ligament reconstruction (ACLR), and to provide a general overview of the statistical performance of these models.

**Methods:**

Three online databases (PubMed, MEDLINE, EMBASE) were searched from database inception to February 6, 2024, to identify literature on the use of machine learning to predict revision, secondary knee injury (e.g. anterior cruciate ligament (ACL) or meniscus), or reoperation in ACLR. The authors adhered to the PRISMA and R-AMSTAR guidelines as well as the Cochrane Handbook for Systematic Reviews of Interventions. Demographic data and machine learning specifics were recorded. Model performance was recorded using discrimination, area under the curve (AUC), concordance, calibration, and Brier score. Factors deemed predictive for revision, secondary injury or reoperation were also extracted. The MINORS criteria were used for methodological quality assessment.

**Results:**

Nine studies comprising 125,427 patients with a mean follow-up of 5.82 (0.08–12.3) years were included in this review. Two of nine (22.2%) studies served as external validation analyses. Five (55.6%) studies reported on mean AUC (strongest model range 0.77–0.997). Four (44.4%) studies reported mean concordance (strongest model range: 0.67–0.713). Two studies reported on Brier score, calibration intercept, and calibration slope, with values ranging from 0.10 to 0.18, 0.0051–0.006, and 0.96–0.97 amongst highest performing models, respectively. Four studies reported calibration error, with all four studies demonstrating significant miscalibration at either two or five-year follow-ups amongst 10 of 14 models assessed.

**Conclusion:**

Machine learning models designed to predict the risk of revision or secondary knee injury demonstrate variable discriminatory performance when evaluated with AUC or concordance metrics. Furthermore, there is variable calibration, with several models demonstrating evidence of miscalibration at two or five-year marks. The lack of external validation of existing models limits the generalizability of these findings. Future research should focus on validating current models in addition to developing new multimodal neural networks to improve accuracy and reliability.

**Supplementary Information:**

The online version contains supplementary material available at 10.1186/s12891-024-08228-w.

## Introduction

The incidence of anterior cruciate ligament (ACL) tears in the United States has been reported at 6.8 per 100,000 annually, making it the most common knee ligament injury [1]. Failure rates amongst primary ACL reconstruction (ACLR) procedures have been estimated to range from 3.2 to 11.1% [2]. Several factors contribute to postoperative failure, including traumatic reinjury, tunnel malposition, and biological failure [3]. Beyond revisions, secondary injuries such as meniscus tears and contralateral injuries are reasons for overall reoperation after primary ACLR [4, 5]. The presence of concomitant meniscal tears, such as ramp lesions with ACL injuries, has been reported to range from 9 to 40% and is associated with a 7.7% rate of secondary meniscectomy [5]. Because of this, it is important for surgeons to have a guide for predicting risk profiles for secondary injury or revision after ACLR.

In recent years, the use of artificial intelligence (AI) has become popular in orthopedic research [5, 6]. Specifically, machine learning (ML) models can learn complex patterns and associations between variables and outcomes from large datasets [7]. These relationships can be used to generate predictive models incorporating patient demographics, injury characteristics, and surgical techniques, which can be used in the clinical setting [4]. Machine learning models can be classified into classical machine learning (Random Forest, Gradient Boosted Regression Model (GBM) etc.) and deep learning with neural networks (NN) (Artificial Neural Networks, Multi-Layer Perceptron etc.). Classical machine learning models tend to be faster and require fewer resources, however, they require manual feature selection, whereas NN models are able to automatically learn features from raw data [8, 9]. ML research has been performed in various orthopedic domains, such as hip arthroplasty, hip arthroscopy, and spinal cord injuries [10–12]. Therefore, the application of ML in predicting objective outcomes following ACLR offers great potential to be used to identify and manage patient expectations, tailor rehabilitation regimens to maximize functional recovery, and to identify optimal candidates for specific surgical interventions.

While the use of AI in ACL literature is promising, surgeons must familiarize themselves with the overall results, advantages, and disadvantages of ML models [13]. To date, no review has provided a comprehensive summary of the utilization of ML models in predicting postoperative outcomes after ACLR. Therefore, this systematic review aims to summarize the statistical performance of machine learning in predicting revision, secondary injury, or reoperations in ACLR, and to provide a general overview of findings from these models. It was hypothesized that ML models would be superior in predicting these outcomes compared to standard logistic regression models.

## Materials and methods

This systematic review was conducted according to the Preferred Reporting Items for Systematic Reviews and Meta-Analyses (PRISMA) and Revised Assessment of Multiple Systematic Reviews (R-AMSTAR) guidelines for coordinating and reporting systematic reviews [14, 15].

### Search strategy

Three online databases (PubMed, Medical Literature Analysis and Retrieval System Online (MEDLINE), Excerpta Medica dataBASE (EMBASE)) were searched from database inception to February 6, 2024, to identify literature on the use of ML to predict revision, secondary injury, or reoperation in ACLR. The search strategy is described in Supplementary Table 1.

Inclusion criteria included the following: (1) studies examining machine learning models to predict objective outcome measures (e.g. revision, reoperation, secondary injury) following ACLR, or external validity of established databases using machine learning models to predict the aforementioned outcomes, (2) simulation-based or laboratory studies and (3) studies written in English. Exclusion criteria included (1) systematic reviews or meta-analyses, (2) text-book chapters, (3) conference abstracts, (4) biomechanical studies, (5) levels of evidence V (i.e. case reports), (6) case series with less than five patients, and (7) cadaveric/animal studies. References of included studies and of pertinent review papers were manually searched to ensure all means of study identification were exhausted. If multiple papers reported the same outcomes using identical patient cohorts, only the article with the largest sample size or latest follow-up period was included. If multiple papers presented overlapping but non-identical cohorts, all articles were included as the extent of patient overlap was unable to be determined.

### Study screening

Two authors independently performed title and abstract screening. Disagreements at this stage were resolved amongst reviewers, and a more senior author was consulted for remaining discrepancies. During the full-text stage, independent screening was performed and conflicts were resolved in a similar fashion.

### Assessment of agreement

The inter-reviewer agreement was evaluated using a kappa (κ) statistic for screening. A priori classification was defined according to the following criteria: a κ of 0.91–0.99 was almost perfect agreement; a κ of 0.71–0.90 was considerable agreement; a k of 0.61–0.70 was high agreement; a κ of 0.41–0.60 was moderate agreement; a κ of 0.21–0.40 was fair agreement and a κ or ICC value of 0.20 or less was no agreement [16].

### Quality assessment

The Methodological Index for Non-Randomized Studies (MINORS) criteria were used for methodological quality assessment [16]. Based on the MINORS criteria, non-comparative studies could get a maximum score of 16. For non-comparative studies, classification was a priori based on a previous systematic review: 0–4 indicated very low-quality evidence, 5–7 indicated low-quality evidence, 8–12 indicated fair-quality evidence, and scores ≥ 13 indicated high-quality evidence [17].

### Data abstraction and outcomes

Two review authors independently extracted and summarized data from included articles using a Google Sheets (Google LLC, Mountain View, CA, USA) spreadsheet. Demographic data such as number of patients, mean age, patient sex, and follow-up times were recorded. Machine learning specifics included the primary outcome of interest (e.g. revision, secondary injury, or reoperation), statistical software used, models assessed, training and test splits, and the handling of missing data. Adherence to the Transparent Reporting of a multivariable prediction model for Individual Prognosis or Diagnosis (TRIPOD) guidelines was also assessed [18].

Discrimination, or classification accuracy, was assessed using area under the receiver operating curve (AUC) and concordance. AUC values range from 0 to 1, with increasing values representing increased discriminatory capacity [19]. Concordance is another representation of AUC, ranging from 0.5 to 1, with increasing scores indicating a model that more accurately identifies the most true positive results and least false negative results [20, 21]. Calibration was assessed using calibration slope, intercept, and error. The calibration intercept is the tendency of a model to overestimate results, with scores approaching 0 indicating less frequent overprediction or underprediction [20, 21]. Calibration slope identifies if predictions are precise or extreme, with scores closer to 1 indicating better model predictions across the range of possible outcomes [20, 21]. Brier scores combine both discrimination and calibration, with values ranging from 0 to 1, with lower scores indicating higher accuracy [22]. Factors deemed highly predictive for revision, secondary injury or reoperation were also extracted. The level of evidence of each paper was reported according to the authors’ statement or, if unstated, was reported using the Oxford Centre for Evidence-Based Medicine (OCEBM) guidelines [23].

### Outcome reporting

Results were presented using descriptive statistics. Means, ranges, percentages, and standard deviations (SD) were calculated using Google Sheets software (Google LLC, Mountain View, CA, USA).

## Results

### Literature search

**Fig. 1:**
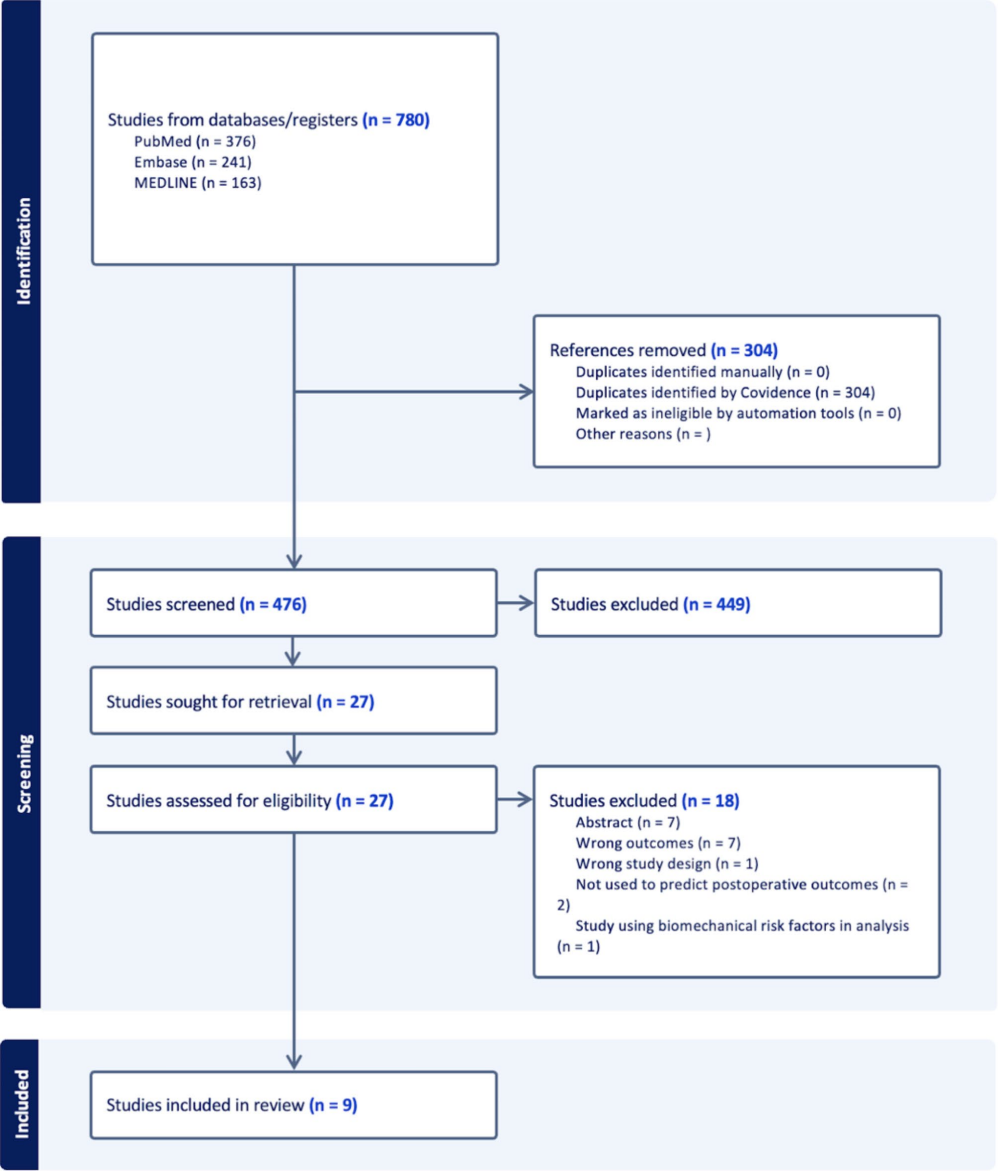
Preferred Reporting Items for Systematic Reviews and Meta-analyses (PRISMA) flow diagram

The initial search resulted in 780 studies, of which 304 were duplicates. Of the 476 remaining, 20 were selected for full-text screening after abstract and title screening. Nine full-text articles satisfied the eligibility criteria and were included in the final analysis (Fig. 1). There was a high level of agreement during title and abstract screening (κ = 0.892, 95%CI 0.799–0.986) and perfect agreement at the full-text stages (κ = 1.00).

### Study quality

All studies included in this review were classified as level IV evidence [24–32]. The mean MINORS score was 11.7 (range: 11–12). On average, the studies included were of fair quality.

### Study characteristics

Five of nine (55.6%) studies reported following the TRIPOD [19] guidelines [24, 25, 27, 29, 30]. Two studies examined the external validity of a previous machine-learning model developed using source data from the Norwegian Knee Ligament Registry (NKLR) database [28, 30]. The other seven studies had their own internal validation [24–27, 29, 31, 32]. Primary model development was performed using the NLKR, DKLR, and Rochester Epidemiology Project databases in three [28, 30, 31], two [30, 31], and three studies [24, 25, 27], respectively. One study each used data from the American College of Surgeons National Surgical Quality Improvement Program database [26], Shanghai Sixth People’s Hospital [32], and the STABILITY I trial [28].

### Demographics

This review included nine studies comprising 125,427 patients with a mean follow-up of 5.82 (range of means: 0.08–12.3) years. Of the eight studies that reported on patient sex, 51,511 female patients (41.5%) were included. The average age of patients at surgery was 26.73 (range of means: 19–32) years. A detailed description of study characteristics and demographics can be seen in Table 1.

**Table 1:** Study characteristics

Author (Year)	Study Design (Level of Evidence)	MINORS SCORE (/16)	Purpose	Source data if external validation study	Database/Institution	TRIPOD Followed?	Number of patients/knees	Mean age at surgery	Female (%)	Follow-up of Outcome (years)
Martin (2023)	Retrospective Cohort (III)	12	Assess sample size effect on accuracy	NR	NKLR + DKLR	Yes	62,955	*MEDIAN* 26 (*IQR: 20–36) [Missing data 1870]*	26,446	7.6 (4.5)
Martin (2022a)	Retrospective Cohort (III)	12	External Validation of Cox Lasso	NKLR	DKLR	Yes	10,922	29 (11)	4916	8.4 (4.3)
Martin (2022b)	Case-control (III)	12	Determine if machine learning analysis of NKLR can identify the most important risk factors associated with subsequent revision of primary ACL reconstruction	NA	NKLR	NR	24,935	28 (11)	10,916	1, 2, 5 years
Johnson (2023)	Retrospective Cohort (III)	12	Machine learning to predict ACL re-operation	NA	Rochester Epidemiology Project	Yes	1400	27	NR	9 years (min 2 years)
Lopez (2023)	Retrospective Comparative Prognostic (IV)	11	Machine learning (ML) models to predict outcomes following ACLR	NR	American College of Surgeons National Surgical Quality Improvement Program database	NR	21,636	31.8 (10.5)	7638 (35.3%)	30 days
Ye (2022)	Case-control (III)	12	Machine learning to determine objective and subjective clinical outcomes of ACLR and to determine the most important predictors	NA	Shanghai Sixth People’s Hospital	NR	432	26.8 (8.4)	112 (25.9%)	6 years (3.1)
Martin (2024)	Retrospective Cohort (III)	11	To assess the external validity of the NKLR model using STABILITY 1 RCT	NKLR	NKLR + STABILITY 1 trial	NR	591	19.0 (3.2)	304 (51.4)	1, 2
Jurgensmeier (2023)	Retrospective Cohort (III)	11	Machine learning to determine risk of secondary meniscal injury post primary ACLR	NA	Rochester Epidemiology Project (REP)	Yes	1187	25 (18–34)	502 (42.3%)	12.3 (6.6–17.6)
Lu (2022)	Retrospective Cohort (III)	12	Machine learning to compare risk and timing of secondary meniscal injury between nonoperative, delayed ACLR, and early ACLR patients	NA	Rochester Epidemiology Project (REP)	Yes	1369	28 (18–37)	677 (40.7)	min 2 year

MINORS: methodological index for non-randomized studies, TRIPOD: transparent reporting of a multivariable prediction model for individual prognosis or diagnosis, NKLR: norwegian knee ligament registry, DKLR: danish knee ligament registry, RCT: randomized controlled trial, NR: not reported, NA: not applicable, ACL: anterior cruciate ligament

### Machine learning methods

Primary outcomes were revision ACLR, secondary meniscus injuries, graft failure, and all-cause reoperation in five [26, 28–31], two [25, 27], one [32], and one study [31], respectively. The most common model was Random Forest/Random Survival Forest, used in six studies [24, 25, 27, 29, 31, 32] (66.6%). The second most used model was the Cox Lasso model, assessed in four studies [28–31] (44.4%). Two studies used neural networks, MLPClassifier (Multi-Layer Perceptron) [24] and Artificial Neural Network (ANN) [26] (22.2%), respectively. With respect to model evaluation, seven (77.8%) studies used calibration [24, 25, 27–31], five (55.6%) used concordance (including C-statistic AUROC) [27–31], four (44.4%) used AUC (including Discrimination - AUROC) [24–26, 32], and two (22.2%) used Brier Score [25, 27].

Five (55.6%) studies used a 75/25 training/test split [24, 28–31], two (22.2%) used 0.632 bootstrapping with 1000 resampled datasets [25, 27], one (11.1%) used an 80/20 training/test split [26], and one (11.1%) used a 90/10 training/test split [32]. Seven studies reported on their handling of missing data [24–27, 29–31], of which five (71.4%) studies used multiple imputation [24, 25, 27, 29, 31]. One external validation study included patients with data for features used in all five models from the source study [30]. Another study excluded patients with missing data [26]. A full description of the Machine Learning Methods can be seen in Table 2.

**Table 2:** Study methods

Author (year)	Primary Outcome	Statistical Software and ML algorithms	Models	Model Evaluation	Training/Test Split	Missing Data Method
Martin (2023)	Revision	R (Version 4.1.11 R Core Team)	Cox lasso Random survival forest Gradient boosting Super learner	Concordance - Harrell C-index Calibration	75/25	Multiple imputation
Martin (2022a)	Revision	R (Version 3.6.1)	Cox Lasso	Concordance - Harrell C-index Calibration	NR (external validation study - original model 75/25)	Patients included if they had data for five predictive models from original model
Martin (2022b)	Revision	R (Version 3.6.1)	Cox Lasso Survival Random Forest Generalized Additive Model (GAM) Gradient Boosted Regression Model (GBM)	Calibration Concordance	75/25	Multiple imputation
Johnson (2023)	All-cause re-operation	SciPy version 1.6.2	MLPClassifier GaussianNB LogisticRegression KNeighborsClassifier BaggingClassifier RandomForestClassifier AdaBoostClassifier GradientBoostingClassifier XGBClassifier	AUC Calibration AUPRC F1 Recall Accuracy Precision	75/25	Multiple imputation
Lopez (2023)	ACLR post-op outcomes (revision included)	TensorFlow Python open-source coding platform (Google Brain, Alphabet Inc., Mountain View, CA)	Artificial Neural Network ML Logistic Regression	AUC Accuracy	80/20	Excluded
Ye (2022)	Graft failure	SPSS (Version 25.0; IBM Corp)	Logistic Regression Gaussian Naïve Bayes Random Forest XGBoost Isotonic XGBoost Sigmoid XGBoost	AUC Accuracy F1	90/10	NR
Martin (2024)	Revision	R (RStudio 2022.07.1)	Cox Lasso	Concordance - Harrell’s C-index Calibration	75/25	NA
Jurgensmeier (2023)	Secondary meniscus tear	R 4.1.2 using RStudio version 1.4.1717 (RStudio, Boston, MA)	SVM Random Forest XGBoost Elastic Net	Discrimination - AUROC Calibration Brier score	0.632 bootstrapping with 1000 resampled datasets	Multiple imputation
Lu (2022)	Secondary meniscus tear	R 4.1.2 using RStudio version 1.2.5001 (RStudio, Boston, MA).	Random Survival Forests	C-statistic (AUROC) (Concordance) Calibration Brier Score	0.632 bootstrapping with 1000 resampled datasets	Multiple imputation

ML: machine learning, AUC: area under the curve, AUROC: area under the receiver operating curve, AUPRC: area under the precision-recall graph, ACLR: anterior cruciate ligament reconstruction, NR: not reported, MA: Massachusetts, CA: California, NR: not reported, NA: not applicable

### Feature selection

Various features were assessed when evaluating machine learning models. Age and body mass index (BMI) were examined in eight [24, 25, 27–32] (88.9%) and six [24–27, 29, 32] (66.7%) studies, respectively. Both sex [24–27, 32] and time between injury and ACLR [28–32] were considered in five (55.6%) studies. Femur fixation method [28–31] and KOOS QOL [28–31] were assessed in four studies each (44.4%), while graft choice [28, 30, 31], sports participation [24, 25, 32], and meniscal injury [29, 31, 32] were examined in three (33.3%) studies each. Other features selected can be seen in Table 3.

**Table 3:** Model Complete Set

Author (year)	Feature Selection	AUC	Calibration Intercept	Calibration Slope	Brier Score	Concordance (95 CI)	Calibration Error
Martin (2023)	Age at surgery Yrs. injury to surgery KOOS QOL Graft: hamstring Age at injury Femur fix: susp/cort. Graft: QT/BQT KOOS Sport Men. injury: none Activity: pivoting Graft: other Fix. comb: susp/interference Surgery on same knee KOOS All low	NR	NR	NR	NR	1 year: Cox Lasso 0.59 (0.56–0.61) RSF: 0.67 (0.64–0.69) GB: 0.67 (0.65–0.70) SL: 0.67 (0.65–0.69) 2 year: Cox Lasso 0.58 (0.56–0.61) RSF: 0.67 (0.64–0.69) GB: 0.67 (0.64–0.69) SL: 0.67 (0.64–0.69) 5 year: Cox Lasso 0.58 (0.56–0.61) RSF: 0.67 (0.65–0.69) GB: 0.67 (0.64–0.69) SL: 0.67 (0.64–0.69)	1 year: Cox Lasso 7.19, n.s RSF: 5.54, n.s GB: 7.48, n.s SL: 8.67, *p* = 0.034 2 year: Cox Lasso 8.17, *p* = 0.043 RSF: 6.42, n.s GB: 4.53, n.s SL: 4.10, n.s 5 year: Cox Lasso: 11.37, *p* = 0.01 RSF: 9.27, *p* = 0.026 GB: 11.07, *p* = 0.011 SL: 11.82, *p* = 0.008
Martin (2022a)	Patient age at primary surgery KOOS QoL score at primary surgery Graft choice Femur fixation method Years between injury and ACLR	NR	NR	NR	NR	1 year: Cox Lasso: 0.678 2 years: Cox Lasso: 0.676 5 years: Cox Lasso : 0.678	1 year: Cox Lasso: 22.24, *p* < 0.001 2 years: Cox Lasso: 11.82, *p* = 0.008 5 years: Cox Lasso : 13.98, *p* = 0.003
Martin (2022b)	Age at surgery Fixation combination Tibia fixation Femur fixation BMI KOOS Sport at surgery KOOS QOL at surgery Years from injury to surgery Age at injury Hospital type Further injury Meniscus injury Injured side	NR	NR	NR	NR	1 year Cox Lasso: 0.686 Random Forest: 0.672 GAM 0.687 GBM 0.669 2 year Cox Lasso 0.684 Random Forest: 0.670 GAM 0.685 GBM: 0.666 5 year: Cox Lasso: 0.683 Random Forest: 0.670 GAM: 0.684 GBM: 0.665	1 year Cox Lasso: 4.89, n.s Random Forest: 3.12, n.s GAM 4.79, n.s GBM 4.98, n.s 2 year Cox Lasso 11.35, *p* = 0.01 Random forest: 11.66, *p* = 0.009 GAM 11.19, *p* = 0.011 GBM: 3.76, n.s 5 year: Cox Lasso: 6.19, n.s Random Forest: 3.71, n.s GAM: 6.98, n.s GBM: 0.38, n.s
Johnson (2023)	Age Sex BMI Occupation Sport participation Injury mechanism Occurrence of reoperation after ACLR	MLPClassifier: AUC = 0.61 GaussianNB: AUC = 0.58 LogisticRegression: AUC = 0.70 KNeighborsClassifier: AUC = 0.68 BaggingClassifier: AUC = 0.75 RandomForestClassifier: AUC = 0.76 AdaBoostClassifier: AUC = 0.73 GradientBoostingClassifier: AUC = 0.75 XGBClassifier: AUC = 0.77	NR	NR	NR	NR	NR
Lopez (2023)	Sex Race BMI (Calculated From The Recorded Height And Weight) American Society Of Anesthesiologists (ASA) Classification History Of Smoking Diabetes Hypertension Requiring Medication Wound Infection Use Of Steroids For A Chronic Condition Bleeding disorders were Abstracted Perioperative Data Such As Anesthesia Type (General, Spinal, IV Sedation, Regional, Other) Surgery Setting (Inpatient Vs Outpatient) Operative Time (Prolonged Operative Time Defined As > 120 min)	ANN: Reoperation: 0.842 ACLR-related Readmission: 0.872 Logistic Regression: Reoperation: 0.601 ACLR-related Readmission: 0.606	NR	NR	NR	NR	NR
Ye (2022)	Age Sex BMI Time From Injury To Surgery Participation In Competitive Sports Preoperative Lysholm And IKDC Scores Posterior Tibial Slope High-Grade Knee Laxity Graft Diameters Of Anteromedial And Posterolateral Bundles Medial And Lateral Meniscal Resection Follow-Up Period Meniscal Reinjury After ACLR	Graft Failure: XGBoost (excellent): AUC = 0.944 (0.001), Accuracy = 0.986 (0.012) Residual Laxity: Random Forest (excellent): AUC = 0.920 (0.014), Accuracy = 0.914 (0.024)	NR	NR	NR	NR	NR
Martin (2024)	Patient Age At Primary Surgery Knee Injury And Osteoarthritis Outcome Score Quality Of Life Subscale (KOOS-QOL) Score At Primary Surgery Graft Choice Femur Fixation Method Time Between Injury And ACLR	NR	NR	NR	NR	1 year: Original Norwegian Algorithm Performance: 0.686 (0.652–0.721) STABILITY data: HT = HT, HT + LET = BPTB: 0.713 (0.634–0.791) HT = HT, HT + LET = Unknown: 0.609 (0.528–0.691) All patients = HT: 0.674 (0.597–07.51) 2 year: Original Norwegian Algorithm Performance: 0.684 (0.650–0.718) STABILITY data: HT = HT, HT + LET = BPTB: 0.713 (0.637–0.789) HT = HT, HT + LET = Unknown = 0.608 (0.530–0.688) All patients = HT: 0.673 (0.598–0.747)	1 year: Original Norwegian Algorithm Performance: 4.9 n.s. STABILITY data: HT = HT, HT + LET = BPTB: 2.6 n.s. HT = HT, HT + LET = Unknown: 10.6 *p* < 0.01 All patients = LT: 8.7 *p* < 0.01 2 year: Original Norwegian Algorithm Performance: 11.3 *p* = 0.01 STABILITY data: HT = HT, HT + LET = BPTB: 11.7 *p* < 0.01 HT = HT, HT + LET = Unknown: 8.9 *p* < 0.01 All patients = LT: 10.2 *p* < 0.01
Jurgensmeier (2023)	Age Sex Body mass index Sport participation Diagnosis of hypermobility or malalignment Radiographic findings Management	SVM: Apparent 0.782 (0.779–0.785), Internal Validation 0.738 (0.736–0.739) Random Forest: Apparent 0.997 (0.994–0.999), Internal Validation 0.790 (0.785–0.795) XGBoost: Apparent 0.814 (0.813–0.816), Internal Validation 0.758 (0.755–0.761) Elastic Net: Apparent 0.673 (0.61–0.736), Internal Validation 0.646 (0.643–0.648)	SVM: 0.0161 (− 0.0173 − 0.0149) Random Forest: 0.006 (0.005–0.0071) XGBoost: 0.007 (0.0055–0.0077) Elastic Net: 0.0165 (0.0152–0.0178)	SVM: 1.091 (1.086–1.096) Random Forest: 0.9608 (0.9562–0.9654) XGBoost: 0.9569 (0.9522–0.9616) Elastic Net: 0.8926 (0.8861–0.8992)	SVM: 0.14 (0.13–0.15) Random Forest: 0.10 (0.09–0.12) XGBoost: 0.12 (0.11–0.14) Elastic Net: 0.18 (0.17–0.20)	NR	NR
Lu (2022)	Age Sex Body mass index Activity level Occupation Comorbid diagnosis Radiographic findings Management	ACLR: 0.80 (0.76–0.83) Non-op: 0.66 (0.58–0.74)	ACLR: 0.0051 (− 0.014–0.024) Non-op: 0.0048 (− 0.059–0.069)	ACLR: 0.97 (0.89–1.05) Non-op: 0.97 (0.65–1.30)	ACLR: 0.106 (0.029–0.183 Non-op: 0.111 (0.034–0.188)	NR	NR

ACLR: anterior cruciate ligament reconstruction, AUC: area under the curve, CI: confidence interval, KOOS: knee osteoarthritis and outcome score, QOL: quality of life, QT: quadriceps tendon, BQT: quadriceps tendon with a bone -block, GB: gradient boosted regression model, RSF: random survival forest, SVM: support vector machine, HT: hamstrings tendon, BPTB: bone-patellar tendon-bone, LET: lateral extra-articular tenodesis, SL: super learner, GAM: generalized additive model, NR: not reported, n.s: not significant, non-op: non-operative

### Model complete set predictive capacity

#### Area under the curve (AUC)

Five studies reported an AUC for their chosen models [24–27, 32]. Overall, AUC for the strongest-performing models in each study ranged from 0.77 to 0.997, indicating that these models ranged from fair to excellent discrimination. The best-performing model was Random Forest (AUC = 0.997) when used to predict secondary meniscus injury [25]. One study found that XGBoost was the best model for predicting graft failure (AUC = 0.944) [32]. When Artificial Neural Network (ANN) was compared with logistic regression, ANN was superior, with an AUC of 0.842 (good discrimination) compared to 0.601 (poor discrimination) for Logistic Regression (LR) [26]. In another study, Random Forest was slightly superior to LR with AUCs of 0.77 and 0.70, respectively [24].

#### Concordance

Four studies reported on concordance [28–31]. Overall, concordance for the best-performing models in each study ranged from 0.67 to 0.71, indicating that these models ranged from poor to fair discrimination. The best-performing model was Cox Lasso (Concordance: 0.71) [28]. One study assessed the STABILITY trial and found that when STABILITY patients with hamstring tendon (HT) autografts in addition to lateral extra-articular tenodesis (LET) were coded as receiving a bone-patellar tendon-bone graft (BPTB) from the NKLR data, this subgroup achieved the highest concordance, with scores of 0.713 (range: 0.634–0.791) and 0.713 (range: 0.64–0.79) at one and two years, respectively [28]. In the same study, the original Norwegian Algorithm reported concordance of 0.686 (range: 0.65–0.72) and 0.684 (range: 0.65–0.72) at one and two years, respectively [28].

One study reported predicting revision concordance for several models over different time intervals. At the one-year interval, the Cox Lasso model had the lowest concordance, with a score of 0.59 (range: 0.56–0.61) and 0.58 (range: 0.56–0.61) at two and five years. The RSF, GB, and SL models all showed higher concordance scores of 0.67 (ranges, RSF: 0.64–0.69, GB: 0.65–0.70, SL: 0.65–0.69), maintaining their scores at two and five years [31]. Another study reported concordance for the Cox Lasso model of 0.678 at one year, 0.676 at two years, and 0.678 at five years [30]. One study found that the GAM model had the highest concordance across all time points (1-year: 0.687, 2-year: 0.685, 5-year: 0.684) [29]. This was followed by Cox Lasso (1-year: 0.686, 2-year: 0.684, 5-year: 0.683), Random Forest (1-year: 0.672, 2-year: 0.670, 5-year: 0.670), and GBM (1-year: 0.669, 2-year: 0.666, 5-year: 0.665) [29].

### Accuracy

#### Brier scores

Two studies reported on Brier Scores [25, 27]. Random Forest was the most accurate model in a study reporting on secondary meniscal injuries, and the studies had Brier scores ranging from 0.10 to 0.18, indicating low deviation of predictions and actual outcomes. One study found that Random Forest was the most accurate, with a Brier score of 0.10 (range: 0.09–0.12) at a mean follow-up of 12.3 (6.6–17.6) years, with key variables being time to return to sport, visual analog scale (VAS) pain score at injury, and time to surgery [25]. Another study predicting secondary meniscal injuries reported a Brier score of 0.106 (range: 0.029–0.183) at a minimum two-year follow-up using the Random Survival Forest model, with key variables being time to return to sport, VAS pain score at injury, and hypermobility [27].

#### Calibration intercept and calibration slope

Two studies looked at calibration intercept and slope [25, 27]. The best-performing models for calibration interval reported scores ranging from 0.0051 to 0.006. The random survival forest model performed best when predicting secondary meniscal injury after ACLR with a score of 0.0051 (− 0.014 to 0.024) at a minimum two-year follow-up [27]. The positive intercept indicates that included ML models tend to underestimate the risk; however, the confidence interval suggests that the systemic underprediction is not statistically significant. The best-performing models for calibration slope reported slopes from 0.96 to 0.97, with Random Survival Forest reporting the highest score (0.97) at a minimum two-year follow-up in a study predicting second meniscal injury [27]. XGBoost was similar with a slope of 0.957 (0.952–0.962) at a mean follow-up of 12.3 (6.6–17.6) years in a study predicting second meniscal injuries [25]. These values suggest that the models used tend to slightly overestimate risk, placing too much importance on predicting features. Overall, both studies revealed that the calibration intercept and slope were most accurate using the predictive features of time to return to sport and VAS pain score.

#### Calibration error

Calibration error was measured in four studies [28–31]. One study reported calibration errors for various models at one-, two-, and five-year marks [31]. At one year, the Cox Lasso, Random Survival Forest (RSF), and Gradient Boosting (GB) models all had non-significant calibration errors, whereas the Super Learner (SL) model demonstrated a calibration error of 8.67 (*p* = 0.034). At two years, the Cox Lasso model showed a significant calibration error of 8.17 (*p* = 0.043). At five years, calibration errors were significant in all models: 11.37 (*p* = 0.01) for Cox Lasso, 9.27 (*p* = 0.026) for RSF, 11.07 (*p* = 0.011) for GB, and 11.82 (*p* = 0.008) for SL. One study reported significant calibration errors for the Cox Lasso model, with errors of 22.24 (*p* < 0.001) at one year, 11.82 (*p* = 0.008) at two years, and 13.98 (*p* = 0.003) at five years [30]. One study found significant miscalibration at two years, with calibration errors of 11.35 (*p* = 0.01) for Cox Lasso, 11.66 (*p* = 0.009) for Random Forest, and 11.19 (*p* = 0.011) for Generalized Additive Model (GAM). None of the models showed significant calibration errors at five years. Another study externally validated the original Norwegian Algorithm using the STABILITY trial. They found that the subgroup (HT + LET patients coded as having BPTB grafts) with the highest concordance had a significant calibration error of 11.7 (*p* < 0.01) at two years [28]. The original Norwegian Algorithm also showed a significant calibration error of 11.3 (*p* = 0.01) at two years. Other subgroups analyzed showed evidence of miscalibration at one and two years, respectively (*p* < 0.01). Complete data from the model set can be seen in Table 3.

### Multiple imputation data

Only one study reported data on multiple imputation analyses [31]. The concordance data from this study was not significantly different (*p* < 0.05) from the original set. The specific data can be seen in Table 4. However, the calibration data revealed an increased statistically significant calibration error in the multiple imputation cohort. At one year, two of the four models showed miscalibration (*p* > 0.05), and at two and five years, all models showed significant miscalibration (*p* < 0.05). The calibration error at one year ranged from 4.17 to 8.35. At two years, it ranged from 8.34 to 8.98; at five years, it ranged from 8.30 to 14.05.

**Table 4:** Multiple Imputation Data

Multiple Imputation Data Set		
Author	Concordance (95 CI)	Calibration
Martin (2023)	1 year: Cox Lasso 0.59 (0.56–0.61) RSF: 0.66 (0.64–0.69) GB: 0.68 (0.65–0.70) SL: 0.67 (0.65–0.70) 2 year: Cox Lasso 0.59 (0.56–0.61) RSF: 0.67 (0.65–0.70) GB: 0.67 (0.65–0.70) SL: 0.67 (0.65–0.70) 5 year: Cox Lasso 0.58 (0.56–0.61) RSF: 0.67 (0.65–0.70) GB: 0.67 (0.65–0.69) SL: 0.67 (0.65–0.70)	1 year: Cox Lasso 8.35, *p* = 0.039 RSF:4.17, *p* = 0.244 GB: 7.57, *p* = 0.056 SL: 7.99, *p* = 0.046 2 year: Cox Lasso 8.81, *p* = 0.032 RSF: 8.96, *p* = 0.030 GB: 8.98, *p* = 0.030 SL: 8.34, *p* = 0.039 5 year: Cox Lasso: 8.30, *p* = 0.040 RSF: 8.95, *p* = 0.030 GB: 11.53, *p* = 0.009 SL: 14.05, *p* = 0.003
**Original Data Set**		
Martin (2023)	1 year: Cox Lasso 0.59 (0.56–0.61) RSF: 0.67 (0.64–0.69) GB: 0.67 (0.65–0.70) SL: 0.67 (0.65–0.69) 2 year: Cox Lasso 0.58 (0.56–0.61) RSF: 0.67 (0.64–0.69) GB: 0.67 (0.64–0.69) SL: 0.67 (0.64–0.69) 5 year: Cox Lasso 0.58 (0.56–0.61) RSF: 0.67 (0.65–0.69) GB: 0.67 (0.64–0.69) SL: 0.67 (0.64–0.69)	1 year: Cox Lasso 7.19, n.s RSF: 5.54, n.s GB: 7.48, n.s SL: 8.67, *p* = 0.034 2 year: Cox Lasso 8.17, *p* = 0.043 RSF: 6.42, n.s GB: 4.53, n.s SL: 4.10, n.s 5 year: Cox Lasso: 11.37, *p* = 0.01 RSF: 9.27, *p* = 0.026 GB: 11.07, *p* = 0.011 SL: 11.82, *p* = 0.008

KOOS: knee osteoarthritis and outcome score, CI: confidence interval, GB: gradient boosted regression model, RSF: random survival forest, SL: super learner, GAM: generalized additive model, n.s: not significant

### Factors predicting outcomes

Various features were considered most important predictive features (top three) by the assessed models. Years from injury to surgery were considered most important by four models (Random Forest, SL, GBM, GAM). Graft choice was considered most important by three models (Cox Lasso, GBM, GAM). Three models considered age at surgery most important (Random Forest, SL, GBM). Femur fixation was considered most important by three models (Cox Lasso, GBM, and GAM). A comprehensive list of the importance of the other features can be seen in Table 5.

**Table 5:** Model performance

Author (year)	Factors predicting outcomes (in order of importance)
Martin (2023)	**Random forest**: age at surgery age at injury years from injury to surgery KOOS QOL **Cox model (lasso)**: femur fix - susp/cort graft qt/BQT fix comb: interfer/susp Graft other Femur fix interf. **Grand boosted regression**: age at surgery years from injury to surgery graft age at injury KOOS QOL **Super learner**: age at surgery years from injury to surgery KOOS QOL Graft hamstring age at surgery
Martin (2022a)	NA - External validation study
Martin (2022b)	**Cox-Lasso**: graft choice femoral fixation KOOS QoL at time of surgery Years from injury to surgery Age at the time of surgery **Random Forest**: age at time of injury tibial fixation device fixation device combination **GAM**: Graft Years from injury to surgery Femur fixation other KOOS QOL at surgery **GBM**: Age at surgery Years from injury to surgery Femur fixation KOOS QOL at surgery
Lopez (2023)	Surgery Setting Operative Time BMI Age Race
Johnson (2023)	Systemic Inflammatory Disease Distal Tear Location Concomitant MCL Repair VAS Proximal Tear Location
Ye (2022)	Medial Meniscal Resection Participation In Competitive Sports Posterior Tibial Slope Graft Diameter Of PLB Male Gender
Martin (2024)	NA - External Validation Study
Jurgensmeier (2023)	Time to RTS VAS Pain Score at injury Time to surgery Age at injury Tear location
Lu (2022)	Time To RTS VAS At Injury Consultation Hypermobility Involvement In Noncontact Sports African American Race

RTS: return to sport, VAS: visual analogue scale, PLB: posterolateral bundle, MCL: medial collateral ligament, BMI: body mass index, KOOS: knee osteoarthritis and outcome score, QOL: quality of life, GBM: gradient boosted regression model, GAM: generalized additive model, NA: not applicable

## Discussion

The primary finding of this systematic review was that existing machine learning models to predict secondary injury or surgery after ACLR are variable in terms of discriminatory performance. Overall, Random Forest models were the most effective at predicting outcomes when using AUC, Brier, Calibration slopes and intercepts. Cox-Lasso was the most effective model when using concordance. Of the four studies reporting on AUC, values were relatively high, ranging from 0.77 to 0.997. However, of the four studies reporting on concordance, the mean values of all studies were closer to 0.5 than 1.0. Furthermore, there was variability when evaluating calibration. While the two studies reporting on Brier scores, calibration slope, and intercept reported minimal evidence of miscalibration in highest performing models, the four studies reporting on calibration error found significant evidence of miscalibration at either two and five-year follow-ups amongst 10 of 14 models assessed. Factors deemed important for secondary ACLR or injury (e.g. secondary meniscus injury, graft failure) were also variable from model to model and study to study.

Machine learning has become incredibly popular in developing models to predict postoperative outcomes, and there is immense potential benefit in using these analyses to generate prediction models and calculators. However, this review demonstrates that there is still room for improvement in model performance. One recent study of 104 patients reported AUC values for several factors predictive of revision, ranging from 0.756 to 0.813 [33]. These values fall in the range of the predictive models that reported on AUC included in this review. Some models in this review had AUCs over 0.95 for predicting secondary meniscus injuries and revision, suggesting strong discriminatory power [25, 32]. However, it is notable that other studies providing C-statistics reported relatively low discrimination, with mean values all being under 0.75. The findings in this review align with a recent systematic review on ML models in various orthopaedic sub-specialties [4]. They found that in spine surgery, hip arthroscopy, total joint arthroplasty, and shoulder arthroplasty, the C-statistics ranged from 0.65 to 0.92, 0.51–0.94, 0.63–0.89, and 0.70–0.95, respectively. While some models from this review had low C-statistics (e.g., closer to 0.5), others had values closer to 1 [4]. They also noted the lack of external validation and inconsistent adherence to predictive modeling guidelines. Therefore, it is possible that existing models investigating revision and secondary injury risk may be missing key important factors.

External validation studies are essential to assess the generalizability of machine learning models. One included study attempted to externally validate the revision prediction model from the original NKLR dataset using the Danish Knee Ligament Registry (DKLR) [30]. Concordance was similar between populations (DKLR: 0.68; NKLR: 0.68–0.69); however, there was significant evidence of miscalibration at one, two, and five years when evaluating the DKLR group. Furthermore, compared to the NKLR dataset, calibration error at one and five years was greater (4.89 versus 22.24 and 6.19 and 13.98 respectively) [29]. The other external validation study assessing the STABILITY trial using the NKLR model reported a concordance of 0.71; however, it found significant evidence of miscalibration at two years. While two of six studies demonstrated strong calibration, these models have not been externally validated like that of the NKLR database. Having models demonstrate strong calibration and concordance at the two-year mark is incredibly important as one in 17 (6%) of ACLR patients will suffer a second ACL injury within two years of the index operation. Furthermore, rates of secondary ACL injury (e.g. ipsilateral or contralateral) at five, ten, and fifteen years have been reported to be 12%, 27%, and 31% [34]. Continuous evaluation of established and novel machine learning algorithms is incredibly important for prediction calculators to translate effectively into clinical practice.

Factors that were not included in the current review that may be important when considering secondary injury risk include concomitant lateral extra-articular tenodesis (LET) procedures, meniscus status, medial collateral ligament (MCL) injuries, and elevated posterior tibial slope (PTS) (or effects of bone morphology). The STABILITY I study, a large multicenter randomized controlled trial (RCT) comparing ACLR with and without LET, found that at 24 months postoperative, the LET group had a rupture rate of 4.1% compared to 11.2% in the non-LET group (*p* = 0.004) [35]. A secondary analysis from this trial demonstrated that younger age, greater posterior tibial slope, high-grade knee laxity, and earlier return to sport all contributed to increased odds of rupture. Larger hamstring autograft diameter was protective in reducing the odds of knee laxity in the form of asymmetric pivot shift [13]. Several of these factors were not a part of the risk calculators developed from machine learning algorithms. Specifically, some studies suggest that an increased PTS may place strain on the ACL, increasing the risk of failure [36]. Certain groups have proposed a threshold of 12 degrees, and have advocated for the use of slope-reducing osteotomies to reduce the PTS, especially in revision settings [37].

Machine learning analyses offer immense potential in terms of predictive capacity, however it is clear that there is much room for improvement, especially in the field of predicting revision or secondary knee injury after ACLR. With the risk of revision still being an issue, this review advocates for including factors such as the inclusion of LET procedures, graft diameter, meniscus status, and elevated posterior tibial slope in developing these models. Furthermore, future studies are encouraged to continue to attempt to externally validate existing and novel models to assess generalizability. Demonstrating strong concordance or AUC and little evidence of miscalibration both in the short-term and long-term is essential in order to implement risk-calculators in the clinical setting. There are a few limitations to this review. First, there were only two inclusions that served as external validation studies, which limit the generalizability of the reported findings. Second, only 55% of studies reported adhering to the TRIPOD guidelines for diagnostic studies, indicating high variability in the quality of individual datasets and reporting of results. This limitation is also noted in a recent systematic review on ML models in orthopaedic trauma, which reported a TRIPOD statement adherence of 62% [38], highlighting the need for better adherence to reporting guidelines. Third, there were limited amounts of comparisons with traditional multivariate logistic regression analyses, preventing the ability to make conclusive statements about the superiority or inferiority of machine learning models when the two methods are compared. Only two studies in this review included NN models, which is another source of weakness. NN modelling would allow for the inclusion of image data and, thus, the creation of multimodal models that incorporate images and clinical variables. In this review, the NNs did not perform better than classical models, which may be because NNs require more resources to create and larger datasets to avoid overfitting. Ultimately, current ACLR prediction models mainly incorporate classical ML, as opposed to multimodel prediction models. Multicenter collaboration based on high-quality prospective databases and registries, with agreement between investigators on feature inclusion, is needed for high-quality ML prediction algorithms. Only nine studies were included in this review, all of which were level IV evidence, preventing the ability to perform a meta-analysis and pool machine learning performance statistics. The average quality of the included studies was fair, which limits the reliability of the findings and highlights the need for further high-quality research in this domain. Finally, it is important to note that these predictive models are preliminary and have not been assessed in a prospective cohort of patients. Future adequately powered longitudinal studies testing these models are needed to ascertain their external validity.

## Conclusion

Machine learning models designed to predict the risk of revision or secondary knee injury demonstrate variable discriminatory performance when evaluated with AUC or concordance metrics. Furthermore, there is variable calibration, with several models demonstrating evidence of miscalibration at two or five-year marks. A key limitation of this study is the lack of external validation of existing models, which restricts their generalizability. Future efforts should focus on validating current models in addition to developing and integrating multimodal neural networks to improve predictive accuracy and reliability. Further comparisons with traditional multivariate logistic regression analysis are also needed to validate the benefit of more advanced models.

## Electronic supplementary material

Below is the link to the electronic supplementary material.

Supplementary Material 1

## Data Availability

Data is provided within the manuscript or supplementary information files.

## Abbreviations

ACL: Anterior cruciate ligament
ACLR: Anterior cruciate ligament reconstruction
AI: Artificial Intelligence
ML: Machine Learning
NN: Neural Networks
PRISMA: Preferred Reporting Items for Systematic Reviews and Meta-Analyses
MINORS: methodological index for non-randomized studies
R-AMSTAR: Revised assessment of multiple systematic reviews
AUC: Area under the curve
OCEBM: Oxford Centre for Evidence-Based Medicine
TRIPOD: Transparent Reporting of a multivariable prediction model for Individual Prognosis or Diagnosis
KOOS QOL: Knee injury and Osteoarthritis Outcome Score Quality of Life
GB: Gradient Boosting
NKLR: Norwegian Knee Ligament Registry
DKLR: Danish Knee Ligament Registry
RSF: Random Survival Forest
RF: Random Forest
LR: Logistic Regression
HT: Hamstring Tendon
LET: Lateral Extra-articular Tenodesis
BPTB: Bone-Patellar Tendon-Bone Graft
ANN: Artificial Neural Network
MLPClassifier: Multi-Layer Perceptron Classifier
GAM: Generalized Additive Model
GBM: Gradient Boosted Regression Model
SL: Super learner
VAS: Visual Analog Scale
