# Supplementary material for: Machine learning models predicting risk of revision or secondary knee injury after anterior cruciate ligament reconstruction demonstrate variable discriminatory and accuracy performance: a systematic review
Source: BMC Musculoskelet Disord. 2025 Jan 4;26:16. doi: 10.1186/s12891-024-08228-w (PMC11699785; doi:10.1186/s12891-024-08228-w)
Supplement: Supplementary file 1 — Supplementary Material 1 [file 12891_2024_8228_MOESM1_ESM.docx]

**SUPPLEMENTARY DIGITAL MATERIAL:**

**Table 1. Search Strategy**

| **PubMed: 376** | **EMBASE: 241** | **MEDLINE[OVID]: 153** |
| --- | --- | --- |
| 1. ACL OR anterior cruciate ligament | 1. ACL OR anterior cruciate ligament | 1. ACL OR anterior cruciate ligament |
| 2. Machine learning OR artificial intelligence OR deep learning | 2. Machine learning OR artificial intelligence OR deep learning | 2. Machine learning OR artificial intelligence OR deep learning |
| 3. 1 OR 2 | 3. 1 OR 2 | 3. 1 OR 2 |
